# Mitigation of Racial and Ethnic Differences in Chlamydia and Gonorrhea Testing

**DOI:** 10.1542/pedsos.2025-000513

**Published:** 2025-08-29

**Authors:** Michelle L. Pickett, Rachel Cafferty, Chella Palmer, T. Charles Casper, Cara Elsholz, Andrea T. Cruz, Kristin S. Stukus, Cynthia J. Mollen, Erin M. Augustine, Jennifer L. Reed, Monika K. Goyal

**Affiliations:** 1 Department of Pediatrics, Medical College of Wisconsin, Milwaukee, Wisconsin; 2 Department of Pediatrics, University of Colorado School of Medicine, Aurora, Colorado; 3 University of Utah School of Medicine, Salt Lake City, Utah; 4 Department of Pediatrics, Baylor College of Medicine, Houston, Texas; 5 Department of Pediatrics, Nationwide Children’s Hospital, Ohio State University College of Medicine, Columbus, Ohio; 6 Department of Pediatrics, Children’s Hospital of Philadelphia, Perelman School of Medicine at the University of Pennsylvania, Philadelphia, Pennsylvania; 7 Department of Pediatrics, Lurie Children’s Hospital, Northwestern University Feinberg School of Medicine, Chicago, Illinois; 8 Department of Pediatrics, Cincinnati Children’s Hospital Medical Center, University of Cincinnati College of Medicine, Cincinnati, Ohio; 9 Department of Pediatrics, Children’s National Hospital, George Washington University, Washington, District of Columbia

## Abstract

**BACKGROUND AND OBJECTIVES:**

Minoritized adolescents have higher proportions of chlamydia (CT) and gonorrhea (GC) testing compared with their white peers. Interventions to mitigate bias in care delivery are needed. The objective of this study was to determine whether electronic health record (EHR)–integrated clinical decision support (CDS) mitigates differential testing of patients based on their race and ethnicity.

**METHODS:**

This planned secondary analysis of a prospective pragmatic parent trial at 6 pediatric emergency departments compared targeted vs universally offered CT/GC testing in patients aged 15–21 years. Adolescents completed a tablet-based sexual health screen to determine CT/GC risk. During the targeted phase, CDS was based on the CT/GC risk assessment. During the universally offered phase, a patient’s desire for CT/GC testing triggered EHR-embedded CDS. Racial and ethnic differences with respect to CT/GC testing were compared between phases using a logistic regression model.

**RESULTS:**

During the baseline phase, almost all racial and ethnic groups were more likely to have CT/GC testing compared with non-Hispanic white patients, with the largest difference in non-Hispanic Black patients (adjusted odds ratio, 2.10; 95% CI, 1.83–2.41). Despite the CDS, there was no change in testing during the targeted phase, meaning differential CT/GC testing in minoritized groups persisted (*P* = .35). However, CDS did mitigate racial and ethnic differences in the universally offered phase, meaning all racial and ethnic groups had CT/GC testing at a similar proportion (*P* = .01).

**CONCLUSIONS:**

Universally offered CT/GC testing using an EHR-embedded CDS can mitigate racial and ethnic differences in CT/GC testing among adolescents in a pediatric emergency department.

## Introduction

The Centers for Disease Control and Prevention (CDC) and US Preventive Services Task Force recommend annual screening for chlamydia (CT) and gonorrhea (GC) in *all* sexually active female individuals younger than 25 years.^1,2^ However, differential testing for CT between Black (66% tested) and white (24% tested) adolescents has been demonstrated in the pediatric primary care setting.^3^ Similarly, among adolescents presenting to the emergency department (ED) with sexually transmitted infection (STI)–related symptoms, nonwhite patients (25%–79%) are more likely to have STI testing compared with white patients (8%–18%).^4,5^ Differential testing by race and ethnicity suggests a potential role of clinician bias in the STI testing approach and may partially explain the disproportionate burden of diagnosed STIs on Black and Hispanic adolescents.

As growing data reveal differential treatment of pediatric ED patients based on their race and ethnicity,^6–11^ interventions to mitigate the impact of clinician bias on health care delivery are urgently needed. Protocolization and clinical decision support (CDS) are effective tools to mitigate racial and ethnic inequities in the burden of STI testing.^12–16^ A standardized or automated approach to STI testing for all sexually active adolescents, regardless of race and ethnicity, may eliminate testing and treatment variation. Our parent study sought to compare 2 different approaches to standardized CT/GC testing, universally offered vs targeted, using electronic health record (EHR)–integrated CDS in pediatric EDs.^17^ Thus, the goal of this secondary analysis was 2-fold. First, we sought to examine clinician CT/GC testing behaviors, with a focus on differences in testing by patient race and ethnicity. Second, we compared differences in the mitigation of racial and ethnic differences in CT/GC testing between an EHR-integrated CDS based on patient-reported sexual health risk (targeted) compared with universally offered testing across multiple pediatric EDs. We hypothesized that the use of a CDS tool would mitigate racial and ethnic differences in CT/GC testing proportions among adolescent patients, with the greatest effect seen during the universally offered approach.

## Methods

### Design

This was a planned secondary analysis of a prospective pragmatic comparative effectiveness trial that compared EHR-embedded CDS for universally offered^18^ vs targeted^19^ CT/GC testing in adolescents.^17^ Please see manuscript by Schmidt et al for a more detailed study design.^17^ Briefly, patients were eligible for the parent study if they were English speaking, aged 15–21 years, and presented to 1 of 6 participating pediatric EDs during the study period (January 1, 2021, to September 30, 2022). Patients presenting for sexual assault/abuse, critical illness, or significant developmental delay precluding survey completion were excluded. The baseline phase (January 1, 2021) included a medical record review of patients meeting eligibility requirements before the introduction of CDS. During the targeted and universally offered phases, ED nursing staff offered a tablet to patients, and patients completed a tablet-based validated sexual health screen,^20^ which identified risk for STIs based on sexual behaviors.^17^ Ideally, the tablets were offered to patients shortly after being placed in an ED room, before clinician evaluation, although this could not be guaranteed. The targeted and universally offered phases were implemented in a stepwise design; thus, the dates of universally offered and targeted phases varied by site. Consent was obtained confidentially on the survey with a waiver of parental consent.

During the universally offered testing strategy, patients were first provided with information on the importance of CT/GC testing and CDC testing recommendations and then offered CT/GC testing during their ED visit. They subsequently completed the sexual health screen on the tablet. During the targeted phase, patients were only offered the tablet-based sexual health screen and did not receive any testing recommendations nor were they asked if they wanted GC/CT testing. Two of the sites already used universal tablet-based screening for suicide risk as the clinical standard. At these sites, after the patient completed the suicide risk screen, age-eligible patients were automatically directed to the study consent page. For the remaining sites, the use of tablet-based screening was a novel approach.

Upon completion, ED clinicians received a CDS alert that enabled direct ordering for CT/GC testing when recommended. Testing was recommended during the targeted phase if the patient scored at high risk (defined as any of the following: any STI-related symptom, ≥1 sexual partner within the last 3 months, no condom use during last intercourse, or a prior history of an STI) or if the patient scored at-risk (sexually active but met none of the high-risk criteria) as determined by the sexual health survey.^17,19,20^ The CDS alerted the clinician of the patient’s risk category and instructed them to discuss and order CT/GC testing if the patient agreed. CT/GC testing was recommended during the universally offered phase of the study if the patient requested it via the tablet by answering “yes” to the question “Do you want to be screened for gonorrhea and chlamydia today?” No other information was provided in the CDS beyond the patient request for testing; the clinician was not made aware of patient’s sexual risk category during the universally offered phase. Clinicians did not have access to patients’ responses to the sexual health survey in either phase to maintain privacy and limit bias. The CDS did not alert the clinician in the following circumstances: (1) in the targeted phase, if the patient was determined to be at low-risk (ie, not sexually active); (2) in the universally offered phase, if the patient declined GC/CT testing; or (3) the patient did not complete the sexual health survey.

### Measures

Participant demographics, including race and ethnicity, were abstracted from the EHR. To ensure adequate sample size in each category for this analysis, race and ethnicity were categorized as the following based on frequency counts providing large enough sample sizes for meaningful comparisons: non-Hispanic white, non-Hispanic Black, non-Hispanic other (non-Hispanic, American Indian/Alaskan; non-Hispanic, Asian; non-Hispanic, multirace; and Non-Hispanic, not otherwise specified), Hispanic white and Hispanic other (Hispanic, American Indian/Alaskan; Hispanic, Asian; Hispanic, Black; Hispanic, multirace; and Hispanic, not otherwise specified). Those with unknown ethnicity were considered non-Hispanic and those with unknown race were excluded from the analysis. When available in the EHR, gender was used. Not all institutions routinely collected patient gender, and in those cases, patient sex was abstracted. Genders other than male and female were categorized as “other” because the gender categories were not consistent across the sites that did collect gender data.

In the targeted and universally offered phases, there was no documentation as to whether a nursing staff member offered a tablet to the patient. Thus, as proxy, consent data were used. The tablet was considered offered if the patient selected either “yes, they consent to participate” or “no, they do not consent to participate” in the survey. This was the first screen the patient saw when they received the tablet. It is possible that some patients may have been offered the tablet but immediately declined without answering the consent question. CDS adherence was defined as the clinician ordering CT/GC testing whey they received a CDS recommending testing. Of note, test results were not included as the purpose of this manuscript was to examine bias with testing.

### Analyses

Racial and ethnic differences in CT/GC testing proportions were compared at baseline and upon implementation of CDS-triggered CT/GC testing in both the targeted and universally offered screening approaches. The primary outcome was CT/GC testing, whereas the secondary outcome was tablet offered. These were chosen as outcome measures as they both have the potential to be directly impacted by bias.

Patient characteristics were summarized by strategy implementation arm using medians and quartiles for continuous variables and n’s and percentages for categorical variables. Race and ethnicity, along with outcomes, were summarized by site, using a Pearson chi-square test of independence to compare the sites.

Primary and secondary outcomes were summarized using descriptive statistics in aggregate and by patient race and ethnicity, age, and gender. A Wilcoxon rank-sum test for continuous variables and a Pearson chi-square test of independence for categorical variables was used to compare those who were and were not offered the tablet. Patients were considered to have been offered the tablet to participate in the sexual health survey if tablet-based consent data (ie, the patient selected “yes” or “no” to the consent question) were available. Racial and ethnic differences with respect to CT/GC testing were compared between phases using a logistic regression model with CT/GC testing as the outcome and race and ethnicity, phase, site, age, gender, and the interaction between race and ethnicity and phase as predictors.

A similar analysis was performed within each intervention period with CT/GC testing as the outcome and site, age, gender, race and ethnicity, tablet offered, and the interaction between race and ethnicity and survey as predictors to assess whether the proportions of testing for each racial category differed between those who were and were not offered a tablet. Of note, tablet-based data from site F during the universally offered phase were missing and thus excluded from analysis. This includes data regarding the tablet being offered and CDS alert during the universally offered phase.

The referent group chosen was non-Hispanic white. We acknowledge automatically using non-Hispanic white as the referent group can perpetuate bias and should not be done without purposeful reason. This decision was made purposefully as our study seeks to explore the impact of bias in medical management. This study was approved through a single institutional review board mechanism. All *P* values were reported based on a 2-sided alternative and considered statistically significant when less than .05. Analyses were performed using SAS 9.4 (SAS Institute, Cary, NC).

## Results

Overall, there were 18 256 patients in the baseline phase, 40 185 in the targeted phase, and 36 302 in the universally offered phase. Characteristics of the study population are presented in Table 1. Most patients were female (57.9%) and non-Hispanic white (34.6%). Proportions of CT/GC testing ranged from 7.4% to 7.7% across all phases and were not significantly different. Site-specific demographics are reported in Supplemental Table 1.

### Racial and Ethnic Differences in Tablet Offering by STI Screening Strategy Phase

Patient demographics for those who were offered a tablet and those who were not are presented in Table 2. In the targeted and universally offered phases combined, patients who were offered the tablet were more likely to be non-Hispanic Black or Hispanic white and less likely to be Hispanic, other. In reference to non-Hispanic white patients, Hispanic white patients had an adjusted odds ratio (a0R) of 1.46 (95% CI, 1.26–1.69) and Hispanic, other patients had an a0R of 1.27 (95% CI 1.11–1.44) after adjusting for suicide screening offered, the interaction between suicide screening offered and race and ethnicity, age, and gender.

Adjusting for the same variables, sites with previously established tablet-based suicide screening had higher odds (a0R, 14.13; 95% CI, 12.9, 15.5) of offering patients the tablet to complete sexual health screening than those who did not. In sites with concurrent suicide screening, all racial and ethnic groups were offered the tablets at similar proportions except Hispanic, other patients, who were less likely to be offered the tablet compared with non-Hispanic white patients (a0R, 0.55; 95% Cl, 0.46–0.65).

### Racial and Ethnic Differences in CT/GC Testing During the Entire Study Period

**FIGURE 1.**
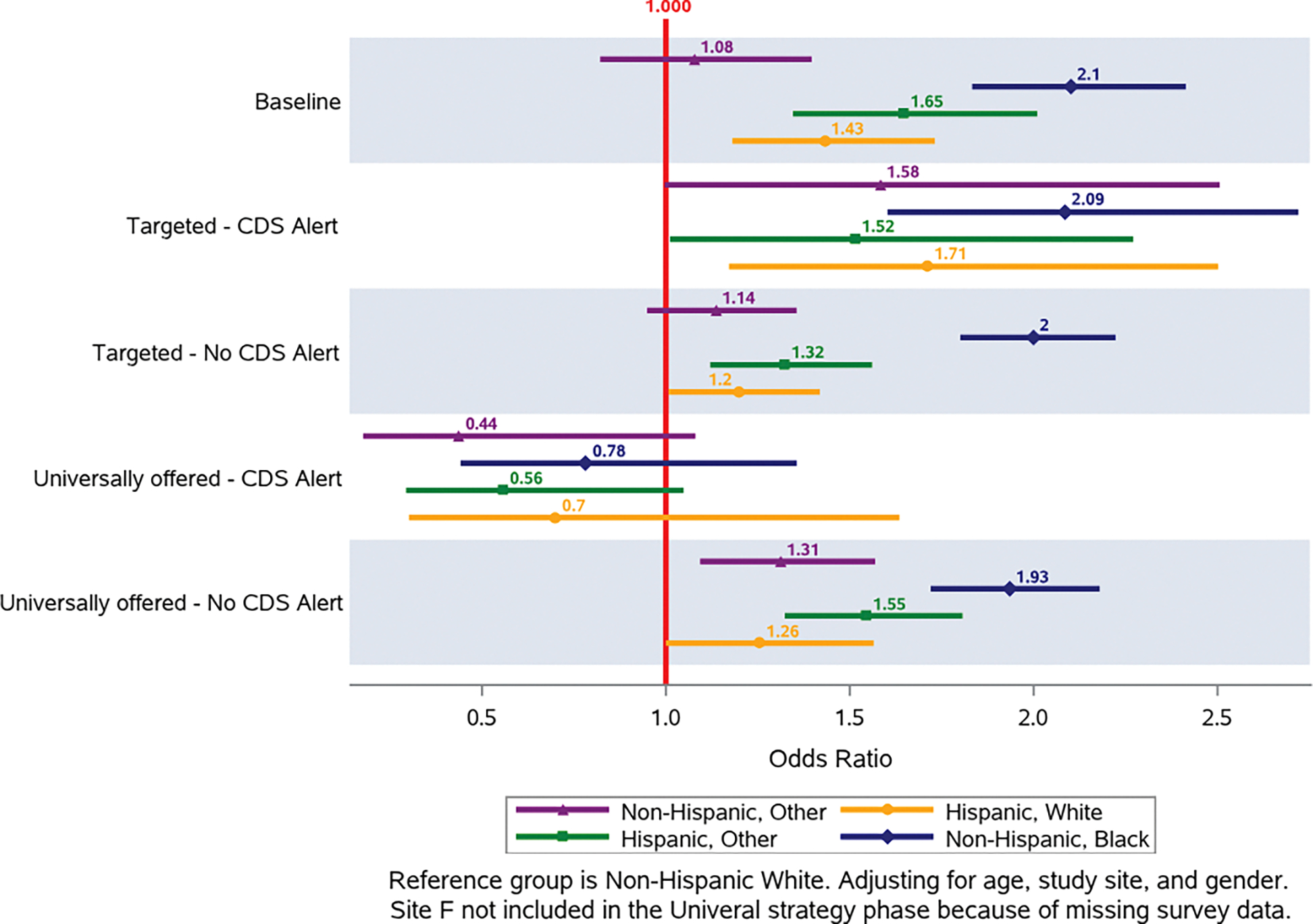
Adjusted odds ratios of differences in chlamydia and gonorrhea testing during the baseline, targeted, and universally offered phases by race and ethnicity. The targeted and universally offered phases show adjusted odds ratios of when the clinician received a CDS alert and when they did not receive a CDS alert. Abbreviation: CDS, clinical decision support.

Demographics of patients who had a CDS alert and clinician adherence to the CDS (defined as CT/GC testing when CDS alerted) are reported in Table 3. Figure 1 demonstrates the ORs by race and ethnicity of CT/GC testing during the baseline phase and among those for whom the CDS did and did not alert in both the targeted and universally offered phases. During the baseline phase, almost all racial and ethnic groups were more likely to undergo CT/GC testing, and non-Hispanic Black patients had the highest odds of having CT/GC testing (a0R, 2.1; 95% CI, 1.83–2.41) compared with non-Hispanic white patients, adjusting for age, site, gender, and the interaction between strategy and race and ethnicity. The interaction between racial and ethnic groups and CDS alerts was not statistically significant (*P* = .35) in the targeted phase, meaning there was no change in testing. The racial and ethnic differences in CT/GC testing persisted and were not mitigated by the CDS alert. Non-Hispanic Black patients continued to have the highest proportion of CT/GC testing (a0R, 2.09; 95% CI, 1.60–2.72).

In the universally offered phase, the CDS alert mitigated all racial and ethnic differences in testing (*P* = .01), meaning there was a change in testing compared with the baseline, and all racial and ethnic groups were tested at similar proportions when the CDS alerted (Figure 1). When the CDS did not alert, racial and ethnic differences in CT/GC testing remained, and almost all racial and ethnic groups were more likely to have CT/GC testing compared with non-Hispanic white patients.

## Discussion

This study provides evidence that racial and ethnic differences in CT/GC testing can be mitigated through a universally offered standardized testing and CDS alert approach. A recently published research agenda calls for an assessment of the impact of standardization in racial inequities in pediatric emergency care,^21^ and our study provides important evidence supporting a standardized approach. Universally offered CT/GC testing is adolescent-driven and based on patient desire, requiring the clinician to merely order a test, remaining unaware of the patient’s STI risk. This approach may decrease the chance that bias, whether unconscious or not, influences the decision to screen. In addition to bypassing clinical judgement, universally offered testing also reduces the clinical effort required by providers by merely ordering the test when requested without personally obtaining a sexual history. Providers, though, should use caution, as other important information, such as pregnancy risk, trafficking risk, and substance use, can be missed if private social histories are not obtained. However, in the targeted phase, racial and ethnic differences in CT/GC testing persisted. In this phase, CT/GC testing was based on the adolescent’s sexual risk, and clinicians were instructed to discuss risk with the patient and subsequently order testing. This approach required more face-to-face interactions with the patient, leading to additional opportunities for unconscious or conscious bias to impact a clinician’s decision to test. Ideally, the patient would have completed the tablet-based screening before clinician evaluation, but in reality, this is not always the case. Oftentimes, additional follow-up with the patient would be required to obtain permission to test, which may limit a clinician’s willingness to take the next step needed to order testing. Additionally, the need to speak privately with the patient about CT/GC testing may contribute to a clinician’s reluctance to test, especially when parents are present, and the presenting symptoms might not be directly related to sexual health.

Our data add to the growing literature in other clinical areas showing that protocolization and CDS are effective tools to mitigate racial and ethnic disparities. The literature has demonstrated a reduction in known racial disparities in maternal and neonatal morbidity rates, cesarean delivery rates, opioid prescribing patterns, and venous thromboembolism prophylaxis among hospitalized patients with protocolization and CDS.^12–16^ Mitigating CT/GC testing differences through a universally offered approach has the potential to impact differences in infection burden. Although universally offered testing does not impact infection in minoritized adolescents directly, it will lead to increased testing in nonminoritized adolescents, which will lead to increased CT/GC detection in this group. Detecting more infections in all adolescents will lessen the racial and ethnic differences in CT/GC burden.

Although it was encouraging that our study eliminated racial and ethnic differences in the universally offered phase of STI testing, there were racial and ethnic variations in who was offered the tablet to complete the sexual health survey, which remains an area of potential bias. These differences persisted despite the use of the tablet-based approach for universal suicide screening at some sites. Overall, Hispanic patients were more likely to be offered a tablet. It is unclear why all patients were not offered a tablet equally, but it could be secondary to bias, specifically toward Hispanic adolescents, highlighting the multiple touchpoints in care in which opportunities for bias exist. Further work, such as automating the provision of the sexual health survey, is needed to eliminate racial and ethnic differences and potential bias. A potential approach includes sending survey links to adolescents’ phones directly upon check-in. However, that requires a minor’s phone numbers to be collected/verified during the registration process just as parent/guardian phone numbers are, which may be challenging in some institutions. It is unknown how bias in who was offered a tablet affects clinician bias in who received a CT/GC test.

Limitations to this study include that the survey was only given to a small proportion of the adolescent population. Additionally, the CDS during the targeted phase may have attenuated racial and ethnic differences if it did not require clinicians to first discuss CT/GC testing with the patient and instead asked patients through the tablet if they wanted to be tested based on their risk status. This would allow the clinician to order the testing, potentially before seeing the patient, and might have removed any bias they had after meeting the patient. Site F was excluded from the universally offered phase owing to missing data, but it had a high proportion of Hispanic patients, which could bias the results. Lastly, race and ethnicity data were abstracted from the EHR instead of self-reported. Although self-reported race and ethnicity are ideal, only EHR data was available during the baseline period, before tablet implementation, and thus, to be consistent across phases, this data source was used for all. Recent literature does demonstrate a high agreement between EHR abstracted and self-reported racial and ethnic data; however, misclassification was highest among some minoritized populations.^22,23^ However, if attempting to understand the role of clinician bias in care delivery, perceived race and ethnicity may be more impactful than self-reported race and ethnicity.

As evidenced by this study and seen in previously published literature, racial and ethnic minoritized adolescents are screened and tested for STIs more often.^3–5,24–26^ This disproportionate and increased testing will lead to increased detection among these groups and thus higher reported rates of STIs among these populations. Higher reported STI rates among racial and ethnic minoritized populations may perpetuate clinician bias and lead to disproportionate and increased STI testing among these populations, which, in turn, will continue the cycle. Differential access to, and provision of, health care services can lead to worse health outcomes, perpetuate bias and stigma, and continue to oppress marginalized populations. One of the overarching goals of Healthy People 2030 is to eliminate health disparities and attain health equity for all.^27^ Over time, universally offered testing may impact disparities in STI rates contributing to the Healthy People 2030 goal to eliminate health disparities.

## Conclusion

We have shown that universally offered testing with a CDS tool can eliminate racial and ethnic differences in STI testing among adolescents in the ED. Although these data are encouraging, they are just a fraction of the equation to combat racial and ethnic disparities in medicine. Larger systemic change, including targeted interventions to diversify the medical workforce, bias and microaggression/microaffirmation training for medical professionals, and policy change, are all needed to improve equity in health and care.

## Supplementary Material

supplementary material

## Figures and Tables

**TABLE 1.** Characteristics of Study Population^a^

	Overall (N = 94 743)	Baseline (n = 18 256)	Targeted (n = 40 185)	Universally Offered (n = 36 302)

Age, median (IQR), years	16.8 (15.9–17.8)	16.8 (15.9–17.8)	16.8 (15.9–17.8)	16.8 (15.9–17.8)
Gender, n (%)^b^				
Female	54 843 (57.9)	10 807 (59.2)	22 979 (57.2)	21 057 (58.0)
Male	39 759 (42.0)	7422 (40.7%)	17 167 (42.7)	15 170 (41.8)
Other^c^	102 (0.1)	20 (0.1)	27 (0.1)	55 (0.2)
Race and ethnicity, n (%)				
Non-Hispanic white	32 763 (34.6)	6947 (38.1)	14 426 (35.9)	11 390 (31.4)
Non-Hispanic Black	30 224 (31.9)	5503 (30.1)	13 263 (33.0)	11 458 (31.6)
Non-Hispanic, other	7663 (8.1)	1225 (6.7)	3355 (8.3)	3083 (8.5)
Non-Hispanic American Indian/Alaskan	200 (0.2)	33 (0.2)	72 (0.2)	95 (0.3)
Non-Hispanic Asian	2361 (2.5)	405 (2.2)	1028 (2.6)	928 (2.6)
Non-Hispanic nultirace	1021 (1.1)	137 (0.8)	480 (1.2)	404 (1.1)
Non-Hispanic, not otherwise specified	4081 (4.3)	650 (3.6)	1775 (4.4)	1656 (4.6)
Hispanic white	11 748 (12.4)	2515 (13.8)	4809 (12.0)	4424 (12.2)
Hispanic, other	12 345 (13.0)	2066 (11.3)	4332 (10.8)	5947 (16.4)
Hispanic, American Indian/Alaskan	66 (0.1)	11 (0.1)	26 (0.1)	29 (0.1)
Hispanic, Asian	44 (0.0)	9 (0.0)	10 (0.0)	25 (0.1)
Hispanic Black	626 (0.7)	103 (0.6)	273 (0.7)	250 (0.7)
Hispanic multirace	578 (0.6)	71 (0.4)	234 (0.6)	273 (0.8)
Hispanic, not otherwise specified	11 301 (11.6)	1872 (10.3)	3789 (9.4)	5370 (14.8)
Study site, n (%)				
A	23 543 (24.8)	3096 (17.0)	12 771 (31.8)	7676 (21.1)
B	15221 (16.1)	1943 (10.6)	5659 (14.1)	7619 (21.0)
C	10 372 (10.9)	2728 (14.9)	2800 (7.0)	4844 (13.3)
D	21 437 (22.6)	5091 (27.9)	8902 (22.2)	7444 (20.5)
E	11 868 (12.5)	2139 (11.7)	5179 (12.9)	4550 (12.5)
F	12 302 (13.0)	3259 (17.9)	4874 (12.1)	4169 (11.5)
Insurance, n (%)				
Commercial	38 887 (41.1)	8303 (45.5)	17 228 (42.9)	13 356 (36.8)
Government	51 353 (54.2)	9145 (50.1)	21 512 (53.6)	20 696 (57.0)
Other	4441 (4.7)	805 (4.4)	1406 (3.5)	2230 (6.1)
STI testing ordered, n (%)	7068 (7.5)	1404 (7.7)	2979 (7.4)	2685 (7.4)
CDS alerted, n (%)^d^	2010 (2.8)	-	1479 (3.7)	531 (1.7)

Abbreviations: CDS, clinical decision support; STI, sexually transmitted infection.

a A total of 3670 were excluded for missing race data.

b Sex was used if gender information was not available.

c Data were only available from 1 site.

d Site F was excluded in the universal phase because of missing survey data.

**TABLE 2.** Demographics of Population Who Were Offered a Tablet to Complete the Sexual Health Survey and Those Who Were Not Offered a Tablet During the Targeted and Universally Offered Phases Combined^a^

	Tablet Offered		
		
	Yes (n = 11 838)	No (n = 60 480)	*P* Value

Age, median (IQR), years	16.7 (15.9–17.6)	16.8 (15.9–17.8)	<.001^b^
Gender, n (%)^c^			<.001^d^
Female	7644 (64.6)	34 134 (56.5)	
Male	4186 (35.4)	26 242 (43.4)	
Other	6 (0.1)	76 (0.1)	
Race and ethnicity, n (%)			<.001^d^
Non-Hispanic white	4125 (34.8)	20 950 (34.6)	
Non-Hispanic Black	4624 (39.1)	19 060 (31.5)	
Non-Hispanic, other^e^	979 (8.3)	5315 (8.8)	
Hispanic white	1181 (10.0)	5848 (9.7)	
Hispanic, other^f^	929 (7.8)	9307 (15.4)	

a Universally offered data for site F were not included because of missing survey data.

b Wilcoxon rank-sum test.

c Sex was used if gender information was not available.

d Pearson chi-square test of independence.

e Includes non-Hispanic, American Indian/Alaskan; non-Hispanic, Asian; non-Hispanic, multirace; and non-Hispanic, not otherwise specified.

f Includes Hispanic, American Indian/Alaskan; Hispanic, Asian; Hispanic, Black; Hispanic, multirace; and Hispanic, not otherwise specified.

**TABLE 3.** Demographics of the Population for Which the Clinician Received a CDS Alert and Adherence to the CDS in Each Targeted and Universally Offered Phase

	Targeted Phase				Universally Offered Phase^a^			
		
	CDS Alert		CDS Adherence		CDS Alert		CDS Adherence	
				
	Yes (n = 1479)	No (n = 38 706)	Yes (n = 787)	No (n = 692)	Yes (n = 531)	No (n = 31 602)	Yes (n = 360)	No (n = 171)

Age, median (IQR), years	17.2 (16.3–18.1)	16.8 (15.9–17.8)	17.3 (16.4–18.2)	17.1 (16.2–18.0)	17.3 (16.3–18.1)	16.8 (15.9–17.8)	17.4 (16.5–18.2)	17.2 (16.1–18.0)
Gender, n (%)^b^								
Female	1022 (69.1)	21 957 (56.7)	577 (73.3)	445 (64.3)	375 (70.9)	18 424 (58.3)	273 (76.0)	102 (60.0)
Male	457 (30.9)	16 710 (43.2)	210 (26.7)	247 (35.7)	151 (28.5)	13 110 (41.5)	83 (23.1)	68 (40.0)
Other	0 (0.0)	27 (0.1)	0 (0.0)	0 (0.0)	3 (0.6)	52 (0.2)	3 (0.8)	0 (0.0)
Race and ethnicity, n (%)								
Non-Hispanic white	448 (30.3)	13 978 (36.1)	223 (28.3)	225 (32.5)	81 (15.3)	10 568 (33.4)	57 (15.8)	24 (14.0)
Non-Hispanic Black	616 (41.6)	12 647 (32.7)	347 (44.1)	269 (38.9)	274 (51.6)	10 147 (32.1)	194 (53.9)	80 (46.8)
Non-Hispanic, other^c^	101 (6.8)	3254 (8.4)	59 (7.5)	42 (6.1)	30 (5.6)	2909 (9.2)	18 (5.0)	12 (7.0)
Hispanic white	172 (11.6)	4637 (12.0)	89 (11.3)	83 (12.0)	39 (7.3)	2181 (6.9)	27 (7.5)	12 (7.0)
Hispanic, other^d^	142 (9.6)	4190 (10.8)	69 (8.8)	73 (10.5)	107 (20.2)	5797 (18.3)	64 (17.8)	43 (25.1)

Abbreviation: CDS, clinical decision support.

Adherence is defined as the clinician ordering a chlamydia/gonorrhea test when recommended by a CDS.

a Site F was not included because of missing survey data.

b Sex was used if gender information was not available.

c Includes non-Hispanic, American Indian/Alaskan; non-Hispanic, Asian; non-Hispanic, multirace; and non-Hispanic, not otherwise specified.

d Includes Hispanic, American Indian/Alaskan; Hispanic, Asian; Hispanic, Black; Hispanic, multirace; and Hispanic, not otherwise specified.

## WHAT’S KNOWN ON THIS SUBJECT:

Minoritized populations are tested at higher proportions for sexually transmitted infections compared with non-Hispanic white patients. Strategies are needed to mitigate the role of clinician bias in racial and ethnic differences in sexually transmitted infection testing rates.

## WHAT THIS STUDY ADDS:

This study adds to the growing literature on standardized, replicable approaches that can mitigate bias in the delivery of care. Clinical decision support can mitigate racial and ethnic differences in chlamydia and gonorrhea testing when offered through a universal approach.

## Abbreviations

a0R: adjusted odds ratio
CDC: Centers for Disease Control and Prevention
CDS: clinical decision support
CT: chlamydia
ED: emergency department
EHR: electronic health record
GC: gonorrhea
STI: sexually transmitted infection

## References

[1] Workowski KA, Bachmann LH, Chan PA, Sexually transmitted infections treatment guidelines, 2021. MMWR Recomm Rep. 2021;70(4):1–187. doi: 10.15585/mmwr.rr7004a1 PMC8344968 34292926

[2] Davidson KW, Barry MJ, Mangione CM, ; US Preventive Services Task Force. Screening for chlamydia and gonorrhea: US Preventive Services Task Force recommendation statement. JAMA. 2021;326(10):949–956. doi: 10.1001/jama.2021.14081 34519796

[3] Wood S, Min J, Tam V, Pickel J, Petsis D, Campbell K. Inequities in *Chlamydia trachomatis* screening between black and white adolescents in a large pediatric primary care network, 2015–2019. Am J Public Health. 2022;112(1):135–143. doi: 10.2105/AJPH.2021.306498 34936422 PMC8713640

[4] Goyal MK, Hayes KL, Mollen CJ. Racial disparities in testing for sexually transmitted infections in the emergency department. Acad Emerg Med. 2012;19(5):604–607. doi: 10.1111/j.1553-2712.2012.01338.x 22594368

[5] Polhemus S, Pickett ML, Liu XJ, Fraser R, Ferguson CC, Drendel AL. Racial disparities in the emergency department evaluation of adolescent girls. Pediatr Emerg Care. 2022;38(7):307–311. doi: 10.1097/PEC.0000000000002675 35353799

[6] Hartford EA, Blume H, Barry D, Hauser Chatterjee J, Law E. Disparities in the emergency department management of pediatric migraine by race, ethnicity, and language preference. Acad Emerg Med. 2022;29(9):1057–1066. doi: 10.1111/acem.14550 35726699

[7] Congdon M, Schnell SA, Londoño Gentile T, Impact of patient race/ethnicity on emergency department management of pediatric gastroenteritis in the setting of a clinical pathway. Acad Emerg Med. 2021;28(9):1035–1042. doi: 10.1111/acem.14255 33745207

[8] Goyal MK, Chamberlain JM, Webb M, ; Pediatric Emergency Care Applied Research Network (PECARN). Racial and ethnic disparities in the delayed diagnosis of appendicitis among children. Acad Emerg Med. 2021;28(9):949–956. doi: 10.1111/acem.14142 32991770

[9] Goyal MK, Johnson TJ, Chamberlain JM, ; Pediatric Care Applied Research Network (PECARN). Racial and ethnic differences in antibiotic use for viral illness in emergency departments. Pediatrics. 2017;140(4):e20170203. doi: 10.1542/peds.2017-0203 28872046 PMC5613999

[10] Goyal MK, Johnson TJ, Chamberlain JM, ; Pediatric Emergency Care Applied Research Network (PECARN). Racial and ethnic differences in emergency department pain management of children with fractures. Pediatrics. 2020;145(5):e20193370. doi: 10.1542/peds.2019-3370 32312910 PMC7193974

[11] Goyal MK, Kuppermann N, Cleary SD, Teach SJ, Chamberlain JM. Racial disparities in pain management of children with appendicitis in emergency departments. JAMA Pediatr. 2015;169(11):996–1002. doi: 10.1001/jamapediatrics.2015.1915 26366984 PMC4829078

[12] Main EK, Chang SC, Dhurjati R, Cape V, Profit J, Gould JB. Reduction in racial disparities in severe maternal morbidity from hemorrhage in a large-scale quality improvement collaborative. Am J Obstet Gynecol. 2020;223(1):123.e1–123.e14. doi: 10.1016/j.ajog.2020.01.026 PMC8923030 31978432

[13] Hamm RF, Srinivas SK, Levine LD. A standardized labor induction protocol: impact on racial disparities in obstetrical outcomes. Am J Obstet Gynecol MFM. 2020;2(3):100148. doi: 10.1016/j.ajogmf.2020.100148 33345879

[14] Grabski DF, Vavolizza RD, Baumgarten HD, Post-operative opioid reduction protocol reduces racial disparity in clinical outcomes in children. J Pediatr Surg. 2024;59(1):53–60. doi: 10.1016/j.jpedsurg.2023.09.030 37858396

[15] Lau BD, Haider AH, Streiff MB, Eliminating healthcare disparities via mandatory clinical decision support: the venous thromboembolism (VTE) example. Med Care. 2015;53(1):18–24. doi: 10.1097/MLR.0000000000000251 25373403 PMC4262632

[16] Charlot M, Stein JN, Damone E, Effect of an antiracism intervention on racial disparities in time to lung cancer surgery. J Clin Oncol. 2022;40(16):1755–1762. doi: 10.1200/JC0.21.01745 35157498 PMC9148687

[17] Schmidt SK, Dexheimer JW, Zorc JJ, Multisite implementation of a sexual health survey and clinical decision support to promote adolescent sexually transmitted infection screening. AppI Clin Inform. 2025;16(2):283–294. doi: 10.1055/a-2480-4628 PMC11964718 39572251

[18] Reed JL, Dexheimer JW, Kachelmeyer AM, Macaluso M, Alessandrini EA, Kahn JA. Information technology–assisted screening for gonorrhea and chlamydia in a pediatric emergency department. J Adolesc Health. 2020;67(2):186–193. doi: 10.1016/j.jadohealth.2020.01.026 32268995 PMC7398829

[19] Goyal MK, Fein JA, Badolato GM, A computerized sexual health survey improves testing for sexually transmitted infection in a pediatric emergency department. J Pediatr. 2017;183:147–152.e1. doi: 10.1016/j.jpeds.2016.12.045 28081888 PMC5440080

[20] Goyal MK, Shea JA, Hayes KL, Development of a sexual health screening tool for adolescent emergency department patients. Acad Emerg Med. 2016;23(7):809–815. doi: 10.1111/acem.12994 27126128 PMC4938750

[21] Portillo EN, Rees CA, Hartford EA, Research priorities for pediatric emergency care to address disparities by race, ethnicity, and language. JAMA Netw Open. 2023;6(11):e2343791. doi: 10.1001/jamanetworkopen.2023.43791 37955894 PMC10644218

[22] Goyal M, Alpern ER, Webb M, ; PECARN IMPROVE and PECARN Registry Study Groups; PECARN IMPROVE and PECARN Registry Study Groups. Agreement of electronic health record-documented race and ethnicity with parental report. Acad Emerg Med. 2024;31(6):613–616. doi: 10.1111/acem.14840 38049203 PMC11147953

[23] Cruz AT, Palmer CA, Augustine EM, Concordance of adolescent gender, race, and ethnicity: self-report versus medical record data. Pediatrics. 2024;153(2):e2023063161. doi: 10.1542/peds.2023-063161 38178777 PMC10827644

[24] Pleasure ZH, Lindberg LD, Mueller J, Frost JJ. Patterns in receipt and source of STI testing among young people in the United States, 2013–2019. J Adolesc Health. 2022;71(5):642–645. doi: 10.1016/j.jadohealth.2022.04.014 35691850

[25] Makrides J, Matson P, Arrington-Sanders R, Trent M, Marcell AV. Disparities in sexually transmitted infection/HIV testing, contraception and emergency contraception care among adolescent sexual minority women who are racial/ethnic minorities. J Adolesc Health. 2023;72(2):214–221. doi: 10.1016/j.jadohealth.2022.08.030 36369111 PMC12513692

[26] Pelliccione A, Modaressi S, Fireman B, Trends in gonorrhea and chlamydia testing and infections across the COVID-19 pandemic in adolescent and young adults in an integrated health system. J Adolesc Health. 2024;75(6):952–957. doi: 10.1016/j.jadohealth.2024.06.029 39152973 PMC11568948

[27] Office of Disease Prevention and Health Promotion. Healthy People 2030 framework. US Department of Health and Human Services. Accessed May 21, 2025. https://odphp.health.gov/healthypeople/about/healthy-people-2030-framework

