# Supplementary material for: Mitigation of Racial and Ethnic Differences in Chlamydia and Gonorrhea Testing
Source: Pediatr Open Sci. Author manuscript; Available in PMC 2026 Jan 21. (PMC12817324; doi:10.1542/pedsos.2025-000513)
Supplement: supplementary material [file NIHMS2131130-supplement-supplementary_material.pdf]

Supplemental Table 1. Racial and ethnic differences for overall, tablet offered and chlamydia (CT)/gonorrhea (GC) testing by site, n (%)

| | | Site | | | | | | |
|---|---|---|---|---|---|---|---|---|
| | Overall | A | B | C | D | E | F | P-value |
| **Overall** | | | | | | | | <.001[1] |
| N per site | 94743 | 23543 | 15221 | 10372 | 21437 | 11868 | 12302 | |
| Non-Hispanic White | 32763 (34.6%) | 7523 (32.0%) | 3829 (25.2%) | 2385 (23.0%) | 12589 (58.7%) | 4102 (34.6%) | 2335 (19.0%) | |
| Non-Hispanic Black | 30224 (31.9%) | 11658 (49.5%) | 2316 (15.2%) | 1952 (18.8%) | 6450 (30.1%) | 4804 (40.5%) | 3044 (24.7%) | |
| Non-Hispanic, Other[2] | 7663 (8.1%) | 2210 (9.4%) | 1600 (10.5%) | 705 (6.8%) | 2077 (9.7%) | 644 (5.4%) | 427 (3.5%) | |
| Hispanic White | 11748 (12.4%) | 409 (1.7%) | 2620 (17.2%) | 245 (2.4%) | 195 (0.9%) | 1917 (16.2%) | 6362 (51.7%) | |
| Hispanic, Other[3] | 12345 (13.0%) | 1743 (7.4%) | 4856 (31.9%) | 5085 (49.0%) | 126 (0.6%) | 401 (3.4%) | 134 (1.1%) | |
| **Tablet offered (targeted/universally offered)[4]** | | | | | | | | <.001[1] |
| N per site | 11838 | 5445 | 708 | 313 | 486 | 4652 | 234 | |
| Non-Hispanic White | 4125 (34.8%) | 1938 (35.6%) | 166 (23.4%) | 66 (21.1%) | 322 (66.3%) | 1587 (34.1%) | 46 (19.7%) | |
| Non-Hispanic Black | 4624 (39.1%) | 2457 (45.1%) | 100 (14.1%) | 58 (18.5%) | 116 (23.9%) | 1837 (39.5%) | 56 (23.9%) | |
| Non-Hispanic, Other[2] | 979 (8.3%) | 562 (10.3%) | 63 (8.9%) | 23 (7.3%) | 46 (9.5%) | 275 (5.9%) | 10 (4.3%) | |
| Hispanic White | 1181 (10.0%) | 107 (2.0%) | 149 (21.0%) | 3 (1.0%) | 0 (0.0%) | 800 (17.2%) | 122 (52.1%) | |
| Hispanic, Other[3] | 929 (7.8%) | 381 (7.0%) | 230 (32.5%) | 163 (52.1%) | 2 (0.4%) | 153 (3.3%) | 0 (0.0%) | |
| **Targeted-CT/GC testing[5]** | | | | | | | | <.001[1] |
| N per site | 2979 | 1179 | 527 | 136 | 553 | 262 | 322 | |
| Non-Hispanic White | 753 (25.3%) | 275 (23.3%) | 114 (21.6%) | 17 (12.5%) | 250 (45.2%) | 48 (18.3%) | 49 (15.2%) | |
| Non-Hispanic Black | 1378 (46.3%) | 733 (62.2%) | 91 (17.3%) | 41 (30.1%) | 248 (44.8%) | 158 (60.3%) | 107 (33.2%) | |
| Non-Hispanic, Other[2] | 209 (7.0%) | 73 (6.2%) | 59 (11.2%) | 10 (7.4%) | 49 (8.9%) | 14 (5.3%) | 4 (1.2%) | |

| | | | | | | | | |
|---|---|---|---|---|---|---|---|---|
| Hispanic White | 323 (10.8%) | 16 (1.4%) | 105 (19.9%) | 3 (2.2%) | 4 (0.7%) | 38 (14.5%) | 157 (48.8%) | |
| Hispanic, Other[3] | 316 (10.6%) | 82 (7.0%) | 158 (30.0%) | 65 (47.8%) | 2 (0.4%) | 4 (1.5%) | 5 (1.6%) | |
| **Universally offered-CT/GC testing[5]** | | | | | | | | <.001[1] |
| N per site | 2685 | 783 | 630 | 298 | 471 | 220 | 283 | |
| Non-Hispanic White | 585 (21.8%) | 143 (18.3%) | 110 (17.5%) | 53 (17.8%) | 192 (40.8%) | 41 (18.6%) | 46 (16.3%) | |
| Non-Hispanic Black | 1171 (43.6%) | 520 (66.4%) | 130 (20.6%) | 73 (24.5%) | 224 (47.6%) | 133 (60.5%) | 91 (32.2%) | |
| Non-Hispanic, Other[2] | 201 (7.5%) | 49 (6.3%) | 73 (11.6%) | 14 (4.7%) | 48 (10.2%) | 9 (4.1%) | 8 (2.8%) | |
| Hispanic White | 272 (10.1%) | 10 (1.3%) | 88 (14.0%) | 8 (2.7%) | 6 (1.3%) | 24 (10.9%) | 136 (48.1%) | |
| Hispanic, Other[3] | 456 (17.0%) | 61 (7.8%) | 229 (36.3%) | 150 (50.3%) | 1 (0.2%) | 13 (5.9%) | 2 (0.7%) | |

[1]Pearson chi-square test of independence

[2]Includes: Non-Hispanic, American Indian/Alaskan; Non-Hispanic, Asian; Non-Hispanic, Multi-race; Non-Hispanic, Not otherwise specified

[3]Includes: Hispanic, American Indian/Alaskan; Hispanic, Asian; Hispanic, Black; Hispanic, Multi-race; Hispanic, Not otherwise specified

[4]Universally offered data for site F not included because of missing survey data

[5]Testing represents all CT/GC testing during the phase regardless tablet offering or survey completion
